# Comparison of a new bioprosthetic mitral valve to other commercially available devices under controlled conditions in a porcine model

**DOI:** 10.1111/jocs.16021

**Published:** 2021-10-05

**Authors:** Dee Dee Wang, Thomas G. Caranasos, Brian P. O'Neill, Richard S. Stack, William W. O'Neill, W. Randolph Chitwood

**Affiliations:** ^1^ Cardiovascular Masters Consortium Durham North Carolina USA; ^2^ Division of Cardiology, Center for Structural Heart Disease Henry Ford Hospital Detroit Michigan USA; ^3^ Division of Cardiothoracic Surgery, Department of Surgery University of North Carolina at Chapel Hill Chapel Hill North Carolina USA; ^4^ Department of Medicine Duke University Durham North Carolina USA; ^5^ Department of Cardiovascular Sciences East Carolina University Greenville North Carolina USA

**Keywords:** epic, left ventricular outflow tract, mitral, mitris, mosaic, valve repair/replacement

## Abstract

**Background/Aim:**

To evaluate three mitral bioprostheses (of comparable measured internal diameters) under controlled, stable, hemodynamic and surgical conditions by bench, echocardiographic, computerized tomography and autopsy comparisons pre‐ and postvalve implantation.

**Methods:**

Fifteen similar‐sized Yorkshire pigs underwent preprocedural computerized tomography anatomic screening. Of these, 12 had consistent anatomic features and underwent implantation of a mitral bioprosthesis via thoracotomy on cardiopulmonary bypass (CPB). Four valves from each of three manufacturers were implanted in randomized fashion: 27‐mm Epic, 27‐mm Mosaic, and 25‐mm Mitris bioprostheses. After CPB, epicardial echocardiographic studies were performed to assess hemodynamic function and define any paravalvular leaks, followed by postoperative gated contrast computerized tomography. After euthanasia, animals underwent necropsy for anatomic evaluation.

**Results:**

All 12 animals had successful valve implantation with no study deaths. Postoperative echocardiographic trans‐valve gradients varied among bioprosthesis manufacturers. The 25‐mm Mitris (5.1 ± 2.7)/(2.6 ± 1.3 torr) had the lowest peak/mean gradient and the 27‐mm Epic bioprosthesis had the highest (9.2 ± 3.7)/(4.6 ± 1.9 torr). Surgical valve opening area (SOA) varied with the 25‐mm Mitris having the largest SOA (2.4 ± 0.15 cm^2^) followed by the 27‐mm Mosaic (2.04 ± 0.23 cm^2^) and the 27‐mm Epic (1.8 ± 0.27 cm^2^) valve. Bench device orthogonal internal diameter measurements did not match manufacturer device size labeling: 25‐mm Mitris (23 × 23 mm), 27‐mm Mosaic (23 × 22 mm), 27‐mm Epic (21 × 21 mm).

**Conclusions:**

Current advertisement/packaging of commercial surgical mitral valves is not uniform. This study demonstrates marked variations in hemodynamics, valve opening area and anatomic dimensions between similar sized mitral bioprostheses. These data suggest a critical need for standardization and close scientific evaluation of surgical mitral bioprostheses to ensure optimal clinical outcomes.

## 1 INTRODUCTION

Surgical valve design has undergone many iterations since 1952, when Charles Hufnagel implanted the first surgical valve, to treat aortic insufficiency.^1^ Valve design has evolved to include development of mechanical, bioprosthetic, and ultimately rapid‐deployment aortic valves for minimally‐invasive approaches. There have also been major advances in the reduced need for anticoagulation, improved hemodynamic performance, and management of patient‐prosthesis mismatch. However, as designs have evolved to tackle these challenges, there has been a lack of direct‐independent scientific comparison of the various bioprosthetic designs.

There is little literature on outcomes of long‐term surgical bioprosthetic mitral valves with regard to echocardiographic gradients, surgical valve true‐annular opening, and true risk of left ventricular outflow tract (LVOT) obstruction. The literature on surgical mitral rings and prosthetic heart valves suggests that manufacturer‐labeled dimensions for surgical mitral bioprostheses are not rooted in scientific data.^2^, ^3^, ^4^ Inconsistent definitions of prosthesis labeled sizing, inconsistences between sizer dimensions and manufacturer labeled valve sizing are complex issues identified by the Valve Labelling Task Force necessitating important regulatory evaluation.^3^ Surgical prosthesis valve sizing and device selection remain not well understood.^3^ Surgical mitral bioprosthetic implantation technique and manufacturer‐issued labeling of device instructions for use vary among vendors. No recent controlled study has evaluated the acute safety, durability, and function of current surgical mitral bioprostheses in a head‐to‐head comparison study. In human clinical trials, this is not feasible due to wide variations in patient‐specific hemodynamic conditions and anatomy, as well as the absence of autopsy verification of in‐situ comparative measurements. This preclinical early feasibility experimental study evaluates three surgical mitral bioprostheses of comparable measured internal diameters in a head‐to‐head study of acute mitral bioprosthetic valve function postimplantation in the setting of controlled anatomical sizing, hemodynamic variables, and surgical expertise.

## 2 MATERIALS AND METHODS

### 2.1 Study design and endpoints

Between August 2020 and January 2021, 15 Yorkshire pigs underwent anatomical evaluation for consideration of enrollment into this study. All of the animals underwent baseline physical screening with on‐site veterinary examination at Synchrony Labs (Synchrony Labs LLC, Durham, North Carolina). This study was supported by Edwards Lifesciences to Synchrony Labs. The funders had no role in the study design, data collection and analysis, decision to publish, or preparation of the manuscript. Study design, evaluation, and implementation was performed by the Cardiovascular Masters Consortium, LLC (CMC). The CMC is an independent group of established physicians in the fields of cardiac surgery and cardiac intervention who objectively assess new cardiovascular technologies using scientifically designed preclinical and clinical studies. The study protocol was approved by the Institutional Animal Care and Use Committee of Synchrony Labs (Synchrony Labs LLC, Durham, North Carolina) and all animals received humane care in compliance with the *Guide for the Care and Use of Laboratory Animals*.^5^

Primary endpoints were defined according to the Mitral Valve Academic Research Consortium criteria for technical, device, and procedural success.^6^ Secondary endpoints evaluated specific device‐related technical failure and complications. This included presence or absence of any paravalvular leak, device positioning, surgical valve opening area (SOA) and bioprosthesis impact on LVOT obstruction(gradient increase ≥10 torr from baseline).^6^ SOA was obtained according to traditional mitral valve leaflet tip area planimetry^7^; defined as the maximal leaflet opening area of the mitral bioprosthesis leaflet tips that corresponded to the largest effective surgical leaflet opening area of the mitral bioprosthesis, obtained on parasternal short axis views by echocardiographic and multiplanar 3D computed tomography (CT) analysis.

### 2.2 Animal preparation and examination

Before procedural consideration, all animals underwent anatomical evaluation with multidetector contrast‐enhanced electrocardiographic (ECG) gated CT scanning, using an on‐site Siemens scanner (Siemens Dual Somatom, Siemens Medical, Forchheim, Germany).^1^, ^8^ Preprocedural screening looked specifically at anatomical characteristics that would be used by a physician in the clinical setting. These data focused on evaluation of subjects' mitral annulus size during maximal diastolic dimensions, left atrial size and trans‐septal catheter crossing height at mid‐end systole. Those with transseptal crossing heights (defined as potential mid‐mid transseptal fossa puncture to mitral annulus distance) ≤15 mm or mitral annulus dimensions (by diameters, area, or perimeter) with greater than 6% variation from other study animals were excluded from enrollment. Twelve pigs, all of similar physical size, met the inclusion criteria.

We performed bench measurements of prosthetic mitral bioprosthesis sizes labeled 25, 27, 29, 31, and 33 mm of the Epic (Abbott, Abbott Park, Illinois), Mosaic (Medtronic, Minneapolis, Minnesota), and Mitris Resilia (Edwards Lifesciences, Irvine, California) valves (Table S1). Inner to inner surgical frame dimensions were captured at multiple levels of each bioprosthesis. Surgical valve size selection for implantation and comparison for this study was determined based on grouping of similar internal surgical frame bench dimensions, and not manufacturer labeled prosthesis sizing. To ensure similar anatomical and hemodynamic study conditions for preclinical evaluation of above surgical valves, pigs with similar sized cardiac anatomy (mitral size, left atrial size, left ventricle [LV] function, and LVOT anatomy) were then identified.

Until the time of valve implantation, the two surgeons were blinded to which surgical valve was being implanted. Surgical valves were randomized between the surgeons to ensure equal opportunity at implantation of all three surgical mitral bioprostheses. Echocardiographic and periprocedural CT imaging was performed by the same imaging physician across all three surgical bioprostheses in all phases of device interrogation. As mentioned earlier, mitral bioprostheses studied were the 27‐mm Abbott Epic, the 27‐mm Medtronic Mosaic, and the 25‐mm Edwards Mitris.

### 2.3 Surgical procedure

Each prosthesis was implanted via a fifth intercostal space left thoracotomy. After systemic heparinization, the descending aorta and right atrium were cannulated for cardiopulmonary bypass (CPB). Then, hypothermic systemic perfusion was established at 28°C. Ventricular fibrillation was induced using a short DC electrical stimulus and maintained throughout by hypothermic perfusion. Through a left atriotomy, the anterior mitral valve leaflet was excised and 12–15 subannular pledgeted 2‐0 Ethibond (Ethicon) sutures were placed circumferentially around the native annulus. Thereafter, the sutures were passed through the prosthesis sewing cuff and the valve was seated. Each suture was secured using titanium Cor‐Knot fasteners (LSI Solutions). Saline ventricular pressurization was done to assure proper valve seating. After the left atrium was closed partially, the left ventricle was deaired and defibrillated. Ventilation was reestablished and the study animal was rewarmed and weaned from CPB. After restoration of normal sinus rhythm and hemodynamic stabilization (similar measurement points), an epicardial 2‐D echocardiographic ultrasound study was done to assess prosthesis transvalvular gradients and ventricular function, as well as to reveal any paravalvular leaks. Each animal was then decannulated and hemostasis was obtained. The thoracotomy was closed in multiple layers and then each anesthetized pig was transported to the on‐site Siemens CT scanner for a postsurgical contrast ECG gated scan to evaluate prosthetic valve function and anatomic orientation.

### 2.4 Data collection and statistics

Periprocedure (anesthetized but unoperated) multidetector retrospectively gated contrast enhanced CT scans were performed on all animals. Upon CT scan completion and dataset acquisition, multiphase cardiac reconstructions of the images were performed at 1.5 mm intervals. Images then were transferred in DICOM (Digital Imaging and Communications in Medicine) format for further evaluation. Postimaging CT processing was performed using Vitrea (Vital Images, Minnetonka, Minnesota) and Mimics (Materialise, Leuven, Belgium) software. All study animals were euthanized and underwent on‐site supervised necropsy with cardiac explantation for anatomical evaluation of each surgical bioprosthesis. Given the small sample size, descriptive data is presented with no further statistical analysis. Continuous and categorical variables are defined as mean and standard deviation; discrete variables are presented as numbers and percentages.^9^

## 3 RESULTS

A total of 15 animals underwent meticulous preclinical CT screening. All animals were screened to obtain accurate size assessments of vascular structures, atria, ventricles, myocardium and mitral annulus. Three animals were excluded due to annular size variations >6%, or transeptal crossing height ≤15 mm as evaluated by CT.

### 3.1 Study population characteristics

For the 12 pigs, baseline age, body weight, left atrial size as well as annular anatomy are depicted in Table 1. There was <6% anatomical variation among all study animals. The mean mitral annulus area was 1410.00 ± 133.60 mm^2^, with mean left atrial height of 28.87 ± 1.93 mm, and mean transseptal crossing height of 20.53 ± 1.48 mm. All pigs randomized to the Mitris device group had a greater frequency of the circumflex artery coursing closely adjacent to the mitral annulus (4/4) versus 2/4 for the Mosaic, and 1/4 in the Epic cohort.

**Table 1:** Baseline porcine demographic information and CT screening anatomical information

	Epic	Mosaic	Mitris
Age at implant (days)	169.8 ± 27.2	165.5 ± 22.8	148.8 ± 7.6
Weight at implant (kg)	88.0 ± 8.95	89.3 ± 6.26	85.0 ± 6.89
Mitral annulus area (sq mm)	1436.75 ± 131.18	1349.0 ± 100.62	1415.25 ± 152.02
Mitral annulus circumference (mm)	139 ± 7.75	133.75 ± 5.38	137.0 ± 7.53
	(vs. Mitris 1.45% variation)	(vs. Mitris 2.40% variation)	
Mitral annulus commissure to commissure distance (mm)	42.7 ± 1.23	40.35 ± 1.31	41.85 ± 2.72
	(vs. Mitris 2.01% variation)	(vs. Mitris 3.65% variation)	
Mitral annulus anterior to posterior distance (mm)	37.35 ± 1.79	38.1 ± 1.0	37.63 ± 2.27
	(vs. Mitris 0.75% variation)	(vs. Mitris 1.24% variation)	
Left atrium width (mm)	43.13 ± 2.79	44.8 ± 1.40	43.0 ± 2.20
	(vs. Mitris 0.30% variation)	(vs Mitris 4.10% variation)	
Left atrium height (mm)	28.35 ± 1.12	29.78 ± 3.17	28.48 ± 1.41
	(vs. Mitris 0.46% variation)	(vs. Mitris 4.46% variation)	
Transseptal crossing height (mm)	19.8 ± 1.49	20.85 ± 1.36	20.93 ± 1.90
(height from a potential mid‐mid transseptal fossa crossing site to the mitral annulus)	(vs. Mitris 5.55% variation)	(vs. Mitris 0.38% variation)	
Frequency of circumflex artery coursing close to mitral annulus	1 out of 4 pigs	2 out of 4 pigs	4 out of 4 pigs

Abbreviation: CT, computed tomography.

Baseline hemodynamic and echocardiographic data were similar among all 12 study animals (Table 2). Mitral valve peak gradients averaged 2.3 ± 0.8 torr, mean gradient 1.1 ± 0.3 torr, and LVOT peak gradients averaged 2.2 ± 0.4 torr (mean 1.1 ± 0.2 torr). All study animals had normal LV function at baseline (Table 2). By echocardiographic evaluation, no animal had underlying pre‐study mitral regurgitation, stenosis, or evidence of left ventricular outflow obstruction.

**Table 2:** Hemodynamics at time of echocardiographic data capture, including baseline echocardiographic measurements

	Epic	Mosaic	Mitris
Baseline			
Systolic blood pressure	102.5 ± 13.77	104.0 ± 11.46	103 ± 11.52
	(vs. Mitris 0.49% variation)	(vs. Mitris 0.97% variation)	
Diastolic blood pressure	57.75 ± 6.65	69.5 ± 7.33	63.75 ± 9.91
Heart rate	73.0 ± 7.44	74.0 ± 8.12	76.25 ± 7.59
	(vs. Mitris 4.36% variation)	(vs. Mitris 3.0% variation)	
Left ventricle ejection fraction	>55%	>55%	>55%
Mitral valve peak gradient (mmHg)	2.6 ± 0.8	2.1 ± 1.0	2.2 ± 0.7
Mitral valve mean gradient (mmHg)	1.2 ± 0.4	1.0 ± 0.3	1.1 ± 0.3
LVOT peak gradient (mmHg)	2.1 ± 0.3	2.3 ± 0.6	2.3 ± 0.5
LVOT mean gradient (mmHg)	1.0 ± 0.2	1.1 ± 0.3	1.2 ± 0.3
Postsurgical valve implant			
Systolic blood pressure	92.5 ± 9.85	91.25 ± 8.81	93 ± 12.65
	(vs. Mitris 0.53% variation)	(vs. Mitris 1.9% variation)	
Diastolic blood pressure	59.0 ± 7.53	64.75 ± 8.73	62.5 ± 8.96
Heart rate	92.5 ± 11.27	89.8 ± 9.29	89.0 ± 10.55
	(vs. Mitris 3.9% variation)	(vs. Mitris 0.89% variation)	

### 3.2 Comparison of valve prosthesis type: major safety, technical, and mechanistic endpoints

#### 3.2.1 Imaging measurements

Postpump acute echocardiographic findings are depicted in Table 2. Among the three studied bioprostheses, in descending order, the 27‐mm Epic mitral bioprosthesis had the highest peak/mean mitral gradient immediately post‐implant, followed by the 27‐mm Mosaic; the 25‐mm Mitris had the least mitral peak/mean gradient (Table 3). Doppler velocity indices parameters of all three mitral prostheses were within normal prosthetic mitral valve function parameters (Table 3).

**Table 3:** Surgical valve opening area by echo and CT versus Doppler parameters of prosthetic mitral valve function (see corresponding Figure 1)

	Epic (27 mm)	Mosaic (27 mm)	Mitris (25 mm)
Mitral valve peak gradient (mmHg)	9.2 ± 3.7	7.2 ± 4.1	5.1 ± 2.7
Mitral valve mean gradient (mmHg)	4.6 ± 1.9	3.9 ± 2.4	2.6 ± 1.3
Mitral valve peak velocity (cm/s)	148.5 ± 32.54	129.5 ± 39.33	99.1 ± 27.53
Mitral valve VTI (cm)	30.38 ± 5.89	26.65 ± 7.79	21.53 ± 6.59
LVOT VTI (cm)	19.6 ± 2.95	21.08 ± 3.58	14.39 ± 5.73
aSurgical valve opening area (cm^2^) by 2D Echo	1.8 ± 0.27	2.04 ± 0.23	2.4 ± 0.15
aSurgical valve opening area (mm^2^) by 3D multiplanar CT	181.5 ± 16.94	206.75 ± 26.6	228.25 ± 12.31

Abbreviation: CT, computed tomography.

a Surgical valve opening area defined as planimetry area at level of bioprosthetic leaflet tips during maximal valve opening.

**Figure 1:**
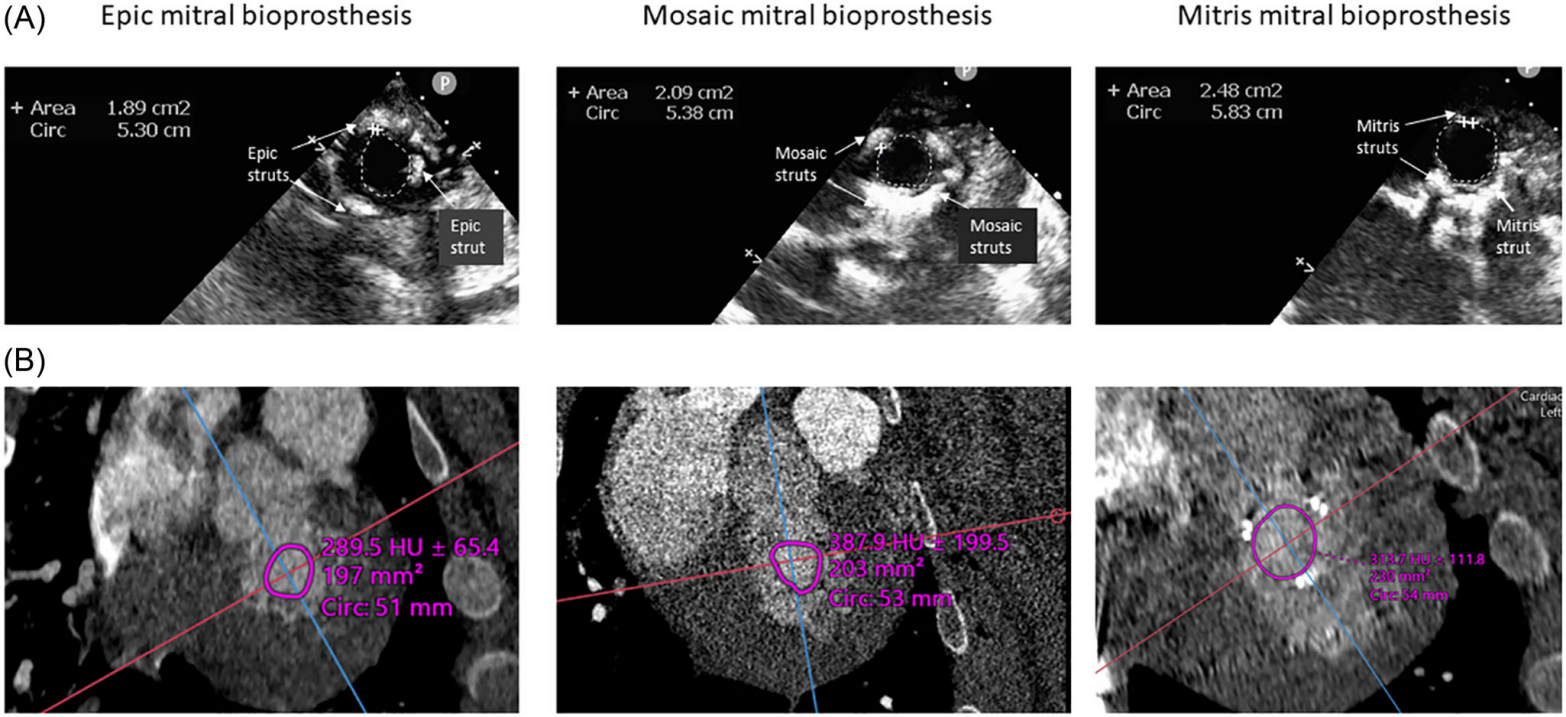
Maximal surgical valve opening area of mitral bioprostheses' leaflets. (A) shows the epicardial 2D echocardiographic planimetry measurement of the surgical mitral bioprosthesis leaflet tips at maximal mid‐end diastolic opening by type of bioprosthesis with trend in ascending order, 27‐mm Epic with the smallest surgical valve opening area, followed by the 27‐mm Mosaic and largest in the 25‐mm Mitris. (B) demonstrates the corresponding maximal mid‐end diastolic mitral bioprosthesis surgical valve opening area leaflet tip planimetry by multiplanar 3D‐CT evaluation with similar trends. CT, computed tomography

The mitral bioprosthesis valve opening area was captured by two independent imaging modalities; multiplanar 3D CT reconstruction and epicardial echo short‐axis view planimetered at the leaflet tips across all 12 surgical cases (Figure 1). Among the three studied bioprostheses, in descending order, the 25‐mm Mitris valve by echocardiographic planimetry had the largest valve opening area (2.4 ± 0.15 cm^2^), followed by the 27‐mm Mosaic (2.04 ± 0.23 cm^2^), and the 27‐mm Epic with smallest valve opening area (1.8 ± 0.27 cm^2^) (Figure 1). These findings were consistent and reproducible as well by the 3D‐CT multiplanar reconstruction post processing evaluations (Table 3 and Figure 1).

**Figure 2:**
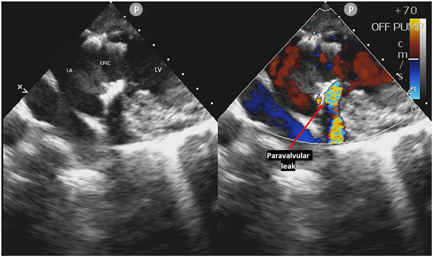
Paravalvular leak in anterolateral trigone of Epic mitral bioprostheses (Video S1). Three of the four Epic mitral bioprostheses were noted to have a paravalvular leak at the anterolateral commissure of the mitral prothesis sewing cuff postcardiopulmonary bypass epicardial echocardiographic interrogation

**Figure 3:**
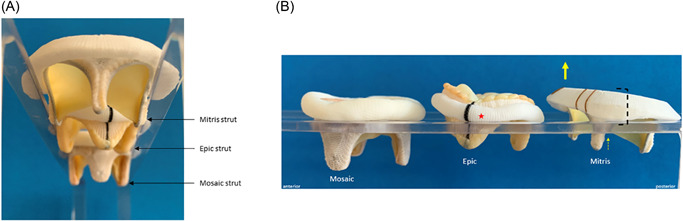
Differences in bioprosthesis strut design. The width of mitral bioprosthesis strut design varies among similar manufacturer labeling “sized” devices. (A) Pictured in the front is the Mitris bioprosthesis with the narrowest strut width, followed by the Epic with the widest strut width, and the Mosaic with the longer strut, but falling in between the Mitris and Epic in strut width. (B) Surgical prosthesis strut length has been long considered a risk factor for turbulent flow and outflow obstruction in the aorta. Side‐by‐side comparison of the Mosaic, Epic and Mitris bioprostheses demonstrate an atrial lift mechanism (yellow arrow) in the anterior arch design (dash bracket) of the Mitris valve that diminishes the amount of strut protruding(dotted yellow arrow) into the aorta despite overall length of the struts

We noted that three of the four Epic bioprostheses had paravalvular annular leak at the anterolateral commissure (Figure 2 and Video S1) (Figure 3 paravalvular leak‐red star) postoperatively following defibrillation and hemodynamic stabilization. All other postimplantation bioprostheses had no central or paravalvular leaks.

#### 3.2.2 Risk of left ventricular outflow tract obstruction measures

There was no clinically significant LVOT obstruction in any study cases. After each successful surgical implantation, a 3D multiplanar CT reconstruction was performed to analyze the depth of the anterolateral and antero‐septal struts within the LVOT (Figure S1). The anterolateral strut of the 27‐mm Mosaic bioprosthesis had the greatest strut depth (mean 11.3 ± 0.94 mm). This was followed by the 25‐mm Mitris device (8.6 ± 0.56 mm) and the 27‐mm Epic with the shortest protrusion (8.4 ± 0.73 mm) (Table 4). This sequence remained similar for the antero‐septal and posterior struts as well.

**Table 4:** Struts versus hemodynamics

	Epic (27 mm)	Mosaic (27 mm)	Mitris (25 mm)
Anteroseptal strut protrusion in LV by CT (mm)	8.2 ± 0.12	11.9 ± 1.03	8.6 ± 0.65
Anterolateral strut protrusion in LV by CT (mm)	8.4 ± 0.73	11.3 ± 0.94	8.6 ± 0.56
Posterior strut protrusion in LV by CT (mm)	9.0 ± 0.87	12.4 ± 0.47	10.0 ± 0.37
LVOT peak gradient	3.4 ± 1.3	4.4 ± 1.3	2.1 ± 1.1
LVOT mean gradient	1.7 ± 0.6	1.9 ± 0.5	1.0 ± 0.6

*Note*: Postsurgical mitral bioprosthesis LVOT obstruction risk evaluation. Postsurgical CT measurements of each bioprosthesis' degree of strut protrusion in the aorta were measured. Shown in Row 4 is the corresponding epicardial echocardiographic LVOT peak to mean gradient for each bioprosthesis subgroup.

Abbreviations: CT, computed tomography; LV: left ventricle, LVOT: left ventricular outflow tract.

The depth of ventricular strut length protrusion did not correlate consistently with postsurgical mitral bioprosthesis LVOT gradients. As mentioned, the 27‐mm Mosaic had the greatest LVOT strut protrusion with the highest peak/mean LVOT gradient (4.4 ± 1.3)/(1.9 ± 0.5 torr). However, despite having the shorter stent frame compared with the 25‐mm Mitris, the 27‐mm Epic trended toward having a higher peak/mean LVOT gradient than its counterpart, which had minimal change in this gradient from baseline (Table 4) (Figure 3).

### 3.3 Bench measurements: bioprosthesis frame internal dimensions

Among the three manufacturers' valves, measured internal diameters of new non‐implanted bioprostheses demonstrated significant differences in valve frame design (Table S1 and Figure S2). For each bioprosthesis, the ratio of the internal diameters of the atrial portion of the device at the level of the sewing ring and ventricular surfaces varied (Figure S2). The 27‐mm Epic device demonstrated an atrial valve internal diameter of 22 mm, which decreased to ventricular internal dimensions of 19 mm. The 27‐mm Mosaic demonstrated similar internal measurements (22 mm) at the atrial portion and decreased to 22 × 21 mm in the ventricular portions. The 25‐mm Mitris internal dimensions were similar within the atrial and ventricular portions of the valve frame design.

Bench‐top true anatomical opening measurements of the Mitris, Epic, and Mosaic valves did not match manufacturer‐labeled numerical designations (Table S1). For the 27‐mm Epic and Mosaic valves, maximum and minimum dimensions were 22 mm. Similar measurements for the 25‐mm Mitris device were 23 mm (internal diameter dimension).

### 3.4 Surgical bioprosthesis strut design in the left ventricular outflow tract

Among the three types of surgical mitral bioprostheses, there was variation in strut length, strut width, and aortic outflow tract diameter opening between struts depending on strut location (Table S2). At the position of the aortic outflow tract, the 27‐mm Mosaic had the longest and widest strut (14‐mm length, 12‐mm width), followed by the 27‐mm Epic (8‐mm length, 11‐mm width) (Table S2). The 25‐mm Mitris had the shortest and narrowest surgical strut (7‐mm length, 4‐mm width). The distance between the anterolateral (“left fibrinous trigone”) and anteroseptal (“right fibrinous trigone”) struts of the bioprostheses oriented to the aortic outflow tract varied depending on the height of the surgical strut protrusion within the aortic outflow tract (Table S2 and Figure S3). At the level of the sewing ring, the 27‐mm Epic device had the smallest aortic outflow tract opening between its anterolateral and anteroseptal struts, measuring 10 mm, as compared to its most ventricular portion measuring 15 mm (Figure S3). The 27‐mm Mosaic had a larger aortic outflow tract distance between its struts at the level of the sewing ring, 12 mm, as compared to its most distal strut markers, 16 mm. The 25‐mm Mitris had the largest aortic outflow opening between its anterolateral and anteroseptal prosthesis struts, measuring 14 and 17 mm, respectively.

## 4 DISCUSSION

Porcine models have been used extensively for evaluation of prosthetic valves.^10^, ^11^, ^12^ This is the first preclinical head‐to‐head evaluation, under controlled anatomic and hemodynamic conditions, of three comparable surgical mitral bioprostheses. This was a fully independent, physician‐designed and ‐executed scientific early feasibility comparison study of multiple current mitral valve bioprostheses.

Here, we show that there was a strong connection between (1) SOA and mitral peak/mean gradient, (2) surgical internal frame design and mitral peak/mean gradient, and (3) width of surgical strut in addition to length of surgical strut for LVOT peak gradients. At the level of the mitral annulus, the 27‐mm Epic had the smallest surgical internal frame dimensions, the smallest surgical valve leaflet tip opening area, the most tapered internal surgical frame design (dimensions extending from the level of the mitral annulus to the level of the distal struts); and the highest peak/mean mitral gradient (9.2 ± 3.7)/(4.6 ± 1.9 torr). The 27‐mm Mosaic had similar internal frame dimensions to the 27‐mm Epic, less tapered internal surgical frame design at the level of the distal struts, and a larger SOA. The 27‐mm Mosaic had a smaller peak/mean mitral gradient, (7.2 ± 4.1)/(3.9 ± 2.4 torr), than the 27‐mm Epic. The 25‐mm Mitris had a larger internal frame dimension, with a nontapered surgical frame design, largest surgical valve leaflet tip opening area, and the smallest peak/mean mitral gradient, (5.1 ± 2.7 torr)/(2.6 ± 1.3 torr), compared with the Epic and Mosaic bioprostheses. Among the three surgical mitral bioprostheses, the 27‐mm Mosaic had the greatest length and greatest width of strut protrusion into the LVOT; and highest peak LVOT gradient (4.4 ± 1.3 torr). The 27‐mm Epic and 25‐mm Mitris devices had comparable strut protrusion lengths into the LVOT (Table 4). However, the 27‐mm Epic had wider strut dimensions (Table S2), and smaller distance between the struts oriented toward the aortic outflow tract, and a higher peak LVOT gradient (3.4 ± 1.3 torr) than the similar length 25‐mm Mitris (2.06 ± 1.05 torr). These data suggest that among similar sized mitral bioprostheses, there is significant variation in bioprosthetic valve form and function.

Selection of prosthetic mitral valve remains difficult.^3^ Labeled prosthetic valve sizing is not standardized.^4^ Internal diameters of surgical valves vary significantly depending on manufacturer, not only at the level of the tissue annulus, but additionally at the level of the ventricular struts.^3^ There are conflicting reports on rates of structural valve deterioration, reoperation, and methodologies on how to assess hemodynamic function between bovine pericardial and porcine stented prosthetic mitral replacement.^13^, ^14^, ^15^, ^16^, ^17^

Given the heterogeneity of mitral bioprosthesis valve design, there is a need for scientific standardization and validation of mitral prostheses' sizing for human clinical implantation. In the clinical context, mitral bioprosthesis valve dysfunction is felt to warrant clinical evaluation for potential future transcatheter mitral valve‐in‐valve replacement (TMVR) therapies once mitral bioprostheses fall to a mitral valve area ≤1.5 cm^2^. A difference of almost 0.6 cm^2^ in SOA in a freshly implanted surgical device without the presence of pannus ingrowth or calcification may account for why some mitral bioprostheses are more commonly associated with need for reoperation or TMVR valve in valve. Additionally, the impact of varying surgical strut design, surgical frame shapes, and width between surgical struts at the anterolateral and anteroseptal trigones may impact the efficacy of potential future transcatheter LVOT modification techniques such as laceration of the anterior mitral leaflet and will need to be further studied.

The decision on selection of a mitral bioprosthesis for a particular patient‐specific anatomy remains a quandary. This study confirmed that manufacturer‐labeled device sizing and strut length protrusion did not match anatomical pathophysiological findings. None of the three surgical mitral bioprostheses ever achieved an internal diameter maximal dimension equivalent to its labeled size. The 27‐mm Epic and Mosaic only had an internal diameter of 22 mm and the 25‐mm Mitris had an internal dimension of 23 mm. The larger ventricular internal dimensions (22 × 21 mm) and SOA (2.04 ± 0.23 cm^2^) of the 27‐mm Mosaic may account for the diminished mitral gradient as compared to the 27‐mm Epic device ([19 mm] and [1.8 ± 0.27cm^2^]). However, the asymmetry of the 27‐mm Mosaic ventricular dimensions and smaller SOA compared to the 25‐mm Mitris (2.4 ± 0.15 cm^2^), may contribute to greater turbulent flow and effectively higher mitral gradients. This study demonstrates that manufacturer‐labeled device sizing number is not consistent across manufacturers and should not be used as a clinical determinant for device size implantation. Thus, knowledge of manufacturers' variations in valve strut width may be helpful to avoid LVOT flow interference.

### 4.1 Limitations

This is a head‐to‐head early feasibility preclinical study on three specific surgical mitral bioprostheses. Several limitations to the study include the small number of animals studied and inability to test all surgical prosthesis sizes to justify certain valve type outcomes. Additionally, this is an acute animal study without ability to evaluate for long‐term mitral bioprosthesis device durability. In this acute animal study, presence or absence of LV remodeling could not be considered in the hemodynamic evaluation of each mitral bioprosthesis. Although all efforts were made to control anatomical and hemodynamic variations, this study serves as a steppingstone for future human clinical studies. The importance of this study demonstrates the need for a pivotal trial with larger number of patients and longer period of follow‐up to thoroughly assess the potential impact of surgical mitral bioprosthesis design on bioprosthesis function. Given the small number of animals in this pilot study, all results should be interpreted as hypothesis generating. Larger studies will be necessary to evaluate for long‐term clinical outcomes.

## 5 CONCLUSIONS

Rigorous scientific evaluation of surgical mitral bioprostheses is necessary for patient safety. Based on these results, we would advise caution when evaluating manufacturers' advertising. Implications of this study demonstrate a critical need for standardization and scientific evaluation of surgical mitral bioprostheses to ensure optimal outcomes for clinical human implantation.

## CONFLICT OF INTERESTS

Dee Dee Wang: Consultant for Edwards Lifesciences, Boston Scientific, Abbott, Neochord. Boston Scientific Research grant support assigned to employer Henry Ford Health System. Member of Cardiovascular Masters Consortium (outside consultants to Synchrony Labs for this study) and Structural Heart Imaging LLC. Thomas G. Caranasos: Member of Cardiovascular Masters Consortium (outside consultants to Synchrony Labs for this study), proctor for Atricure, proctor for Cryolife. Brian P. O'Neill: Consultant to and receives research support from Edwards Lifesciences. Member of Cardiovascular Masters Consortium (outside consultants to Synchrony Labs for this study). Richard S. Stack: Member of Cardiovascular Masters Consortium (outside consultants to Synchrony Labs for this study), Synchrony Labs (a wholly owned subsidiary of Synecor LLC), Structural Heart Imaging LLC. William W. O'Neill: Member of Cardiovascular Masters Consortium (outside consultants to Synchrony Labs for this study), Synchrony Labs (a wholly owned subsidiary of Synecor LLC), Structural Heart Imaging LLC. W. Randolph Chitwood: Consultant for Neochord, Medtronic. Member of Cardiovascular Masters Consortium (outside consultants to Synchrony Labs for this study).

## AUTHOR CONTRIBUTIONS

**Dee Dee Wang, Thomas G. Caranasos, Brian P. O'Neill, Richard S. Stack, William W. O'Neill, and W. Randolph Chitwood**: contributed to the concept/design, data analysis/interpretation, drafting of the manuscript, critical revision, and approval of article.

## Supporting information

Supporting information.

Supporting information.

Supporting information.

Supporting information.

Supporting information.
