# Supplementary figures and images for: Comparison of a new bioprosthetic mitral valve to other commercially available devices under controlled conditions in a porcine model
Source: J Card Surg. 2021 Oct 5;36(12):4654–62. doi: 10.1111/jocs.16021 (PMC9292040; doi:10.1111/jocs.16021)

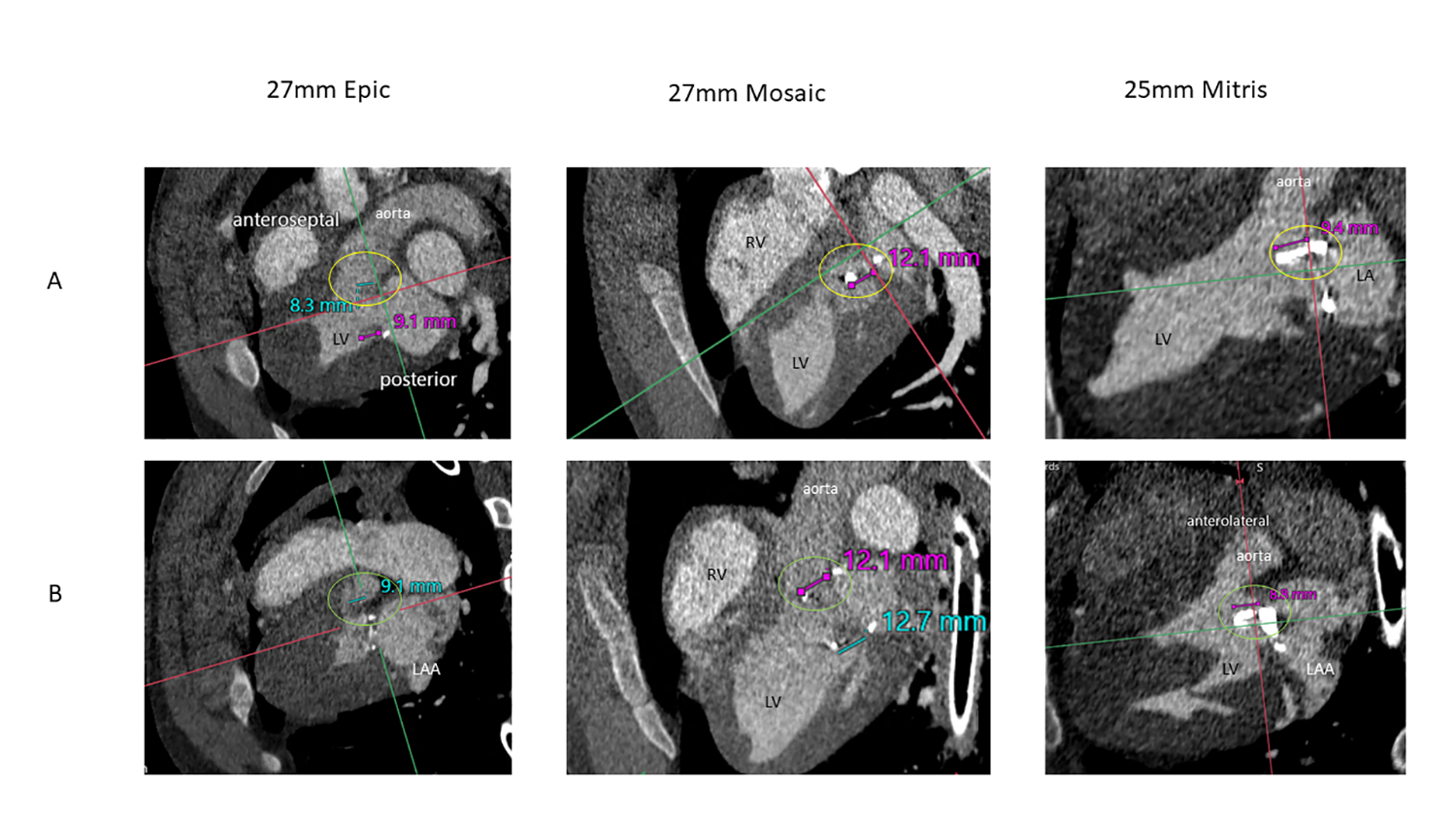

Supplement: Supplementary file 1 — Supporting information. [file JOCS-36-4654-s003.tif]

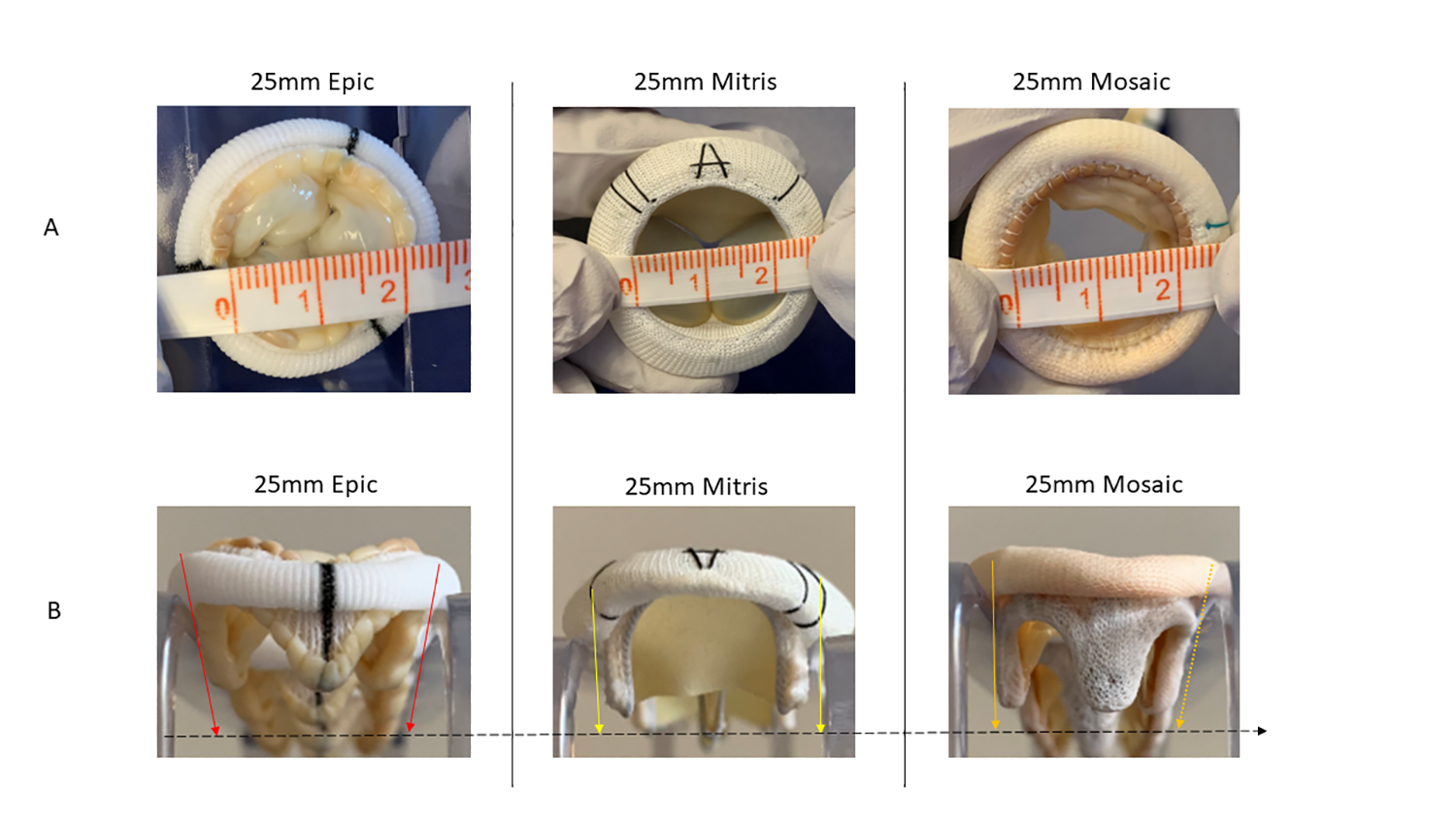

Supplement: Supplementary file 2 — Supporting information. [file JOCS-36-4654-s005.tif]

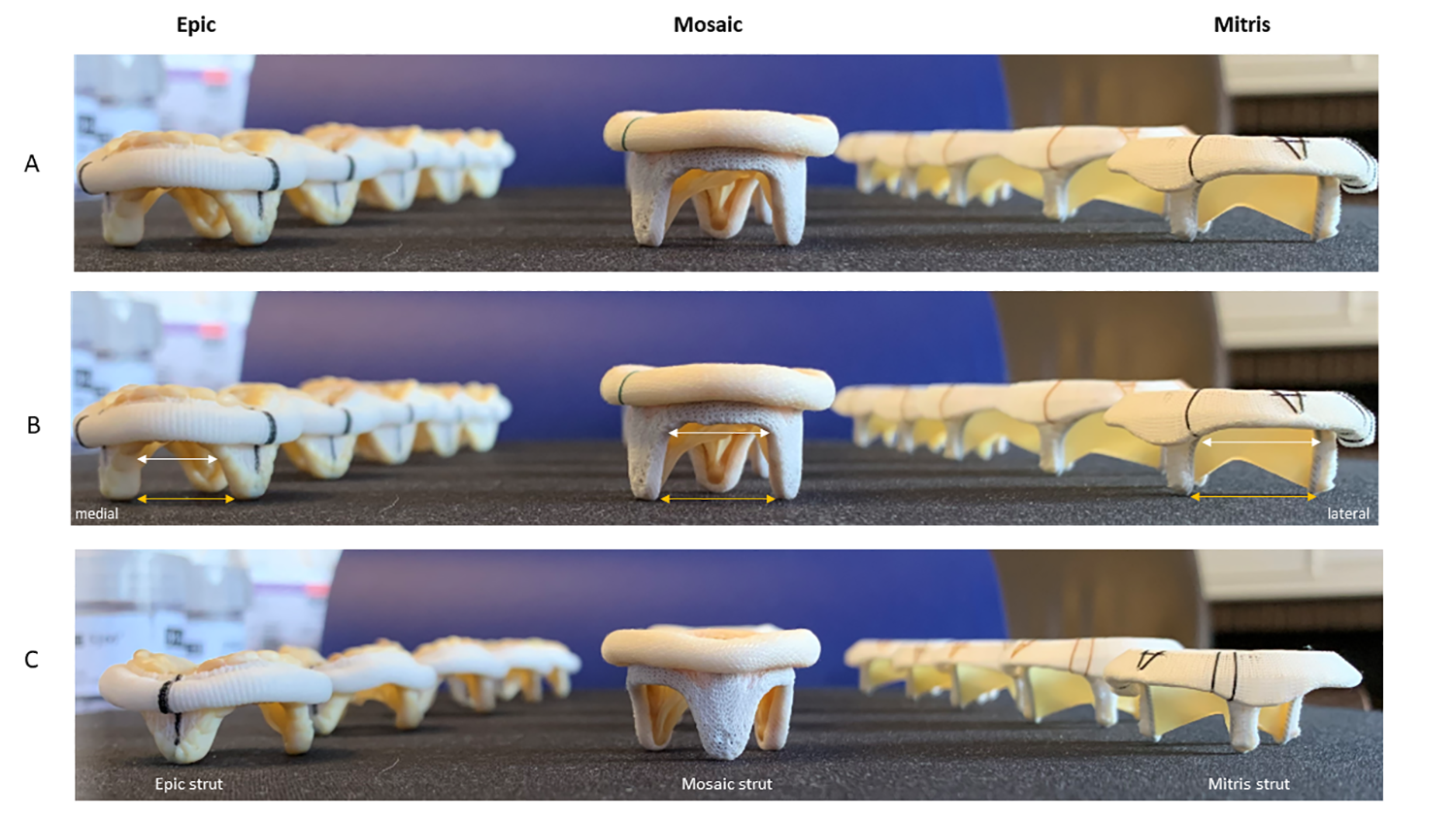

Supplement: Supplementary file 3 — Supporting information. [file JOCS-36-4654-s001.tif]
