# Supplementary material for: Comparison of a new bioprosthetic mitral valve to other commercially available devices under controlled conditions in a porcine model
Source: J Card Surg. 2021 Oct 5;36(12):4654–62. doi: 10.1111/jocs.16021 (PMC9292040; doi:10.1111/jocs.16021)
Supplement: Supplementary file 5 — Supporting information. [file JOCS-36-4654-s004.docx]

**Supplemental Table 1.** Mitral bioprosthesis manufacturer labeled sizing number versus bench inner dimension actual measured sizes. See corresponding Supplemental Figure 1.

|  | **Sewing cuff surgical internal frame dimensions**  **maximum x minimum (mm)** | | |
| --- | --- | --- | --- |
|  | **Epic** | **Mitris** | **Mosaic** |
| **Manufacturer Prosthetic Valve Labeled Sizing** | Maximal x minimal diameter (mm) | Maximal x minimal diameter (mm) | Maximal x minimal diameter (mm) |
| **25** | 20 x 20 | 23 x 23 | 20 x 20 |
| **27** | 22 x 22 | 25 x 25 | 22 x 22 |
| **29** | 23 x 23 | 27 x 27 | 24 x 24 |
| **31** | 25 x 25 | 28 x 28 | 26 x 26 |
| **33** | 28 x 28 | 29 x 29 | 28 x 28 |
|  | **Ventricular surgical internal frame dimensions**  **maximum x minimum (mm)** | | |
|  | **Epic** | **Mitris** | **Mosaic** |
| **Manufacturer Prosthetic Valve Labeled Sizing** | Maximal x minimal diameter (mm) | Maximal x minimal diameter (mm) | Maximal x minimal diameter (mm) |
| **25** | 17 x 17 | 23 x 23 | 20 x 19 |
| **27** | 19 x 19 | 25 x 25 | 22 x 21 |
| **29** | 20 x 20 | 27 x 27 | 24 x 23 |
| **31** | 23 x 23 | 28 x 28 | 25 x 24 |
| **33** | 26 x 26 | 29 x 29 | 26 x 25 |

**Supplemental Table 2.** Bench measurements of different bioprostheses’ strut design. Evaluation of aortic strut protrusion and left ventricular outflow tract prosthesis design.

|  | **Strut length protruding in LVOT “aortic protrusion” (mm)** | | |
| --- | --- | --- | --- |
|  | **Epic** | **Mitris** | **Mosaic** |
| **Manufacturer Prosthetic Valve Labeled Sizing** | **Anteroseptal strut \| Anterolateral strut (mm)** | **Anteroseptal strut \| Anterolateral strut (mm)** | **Anteroseptal strut \| Anterolateral strut (mm)** |
| **25** | 8 \| 8 | 7 \| 7 | 13 : 13 |
| **27** | 8 \| 8 | 9 \| 9 | 14 : 14 |
| **29** | 9 \| 9 | 10 \| 10 | 15 : 15 |
| **31** | 9 \| 9 | 10 \| 10 | 17 : 17 |
| **33** | 9 \| 9 | 10 \| 10 | 17 : 17 |
| **Manufacturer Prosthetic Valve Labeled Sizing** | **Maximal width of strut exposed in LVOT (mm)** | | |
| **25** | 10 | 4 | 12 |
| **27** | 11 | 6 | 12 |
| **29** | 12 | 6 | 13 |
| **31** | 13 | 6 | 13 |
| **33** | 16 | 7 | 15 |
| **Manufacturer Prosthetic Valve Labeled Sizing** | **Distance in mm between struts straddling LVOT at level of**  **(sewing ring within the LVOT) : (most ventricular portion in the LVOT)** | | |
| **25** | 10 : 14 | 14 : 17 | 12 : 16 |
| **27** | 10 : 15 | 16 : 19 | 14 : 17 |
| **29** | 11 : 16 | 18 : 20 | 15 : 18 |
| **31** | 13 : 18 | 19 : 21 | 17 : 20 |
| **33** | 15 : 20 | 20 : 22 | 18 : 22 |

**Supplemental Figure 1. Post-surgical CT aortic strut protrusion.** Row A shows the measurements taken at mid-end systole of the cardiac cycle of the post-surgical CT of the anteroseptal strut length(yellow ellipse highlights measurement of interest) protruding into the left ventricular outflow tract(LVOT). Distance is measured from the level of the mitral annulus to the most ventricular portion of the surgical strut. Row B shows the similar measurement taken across the different mitral bioprostheses of the anterolateral strut length protruding into the LVOT(green ellipse highlights measurement of interest). LAA: left atrial appendage, LV: left ventricle, LA: left atrium, RV: right ventricle

**Supplemental Figure 2. Bench top evaluation of different mitral bioprostheses.** Row A: Shown are the surgeon’s view of the 25-mm Epic, Mitris and Mosaic bioprostheses. Measurements of the inner effective surgical valve opening maximal and minimal diameters were measured across all manufacturer-labeled sizing for each bioprosthesis. Row B: Measurements of the inner surgical prosthesis opening area were additionally obtained at the most ventricular portion of the bioprostheses’ struts. The Epic surgical mitral bioprosthesis demonstrated a larger inner diameter opening area at the level of the atrial sewing cuff as compared to the ventricular surgical opening dimensions; red arrows demonstrate the observed tapering in size of effective surgical frame opening area. The Mitris bioprosthesis demonstrated tubular shape with similar atrial and ventricular surgical inner frame dimensions(yellow arrows). The Mosaic mitral bioprostheses demonstrated asymmetric diameters in the ventricular inner frame with a maximal to minimal dimension difference of 1mm(dotted orange arrow). Dotted horizontal black arrow is a line of reference to visually demonstrate differences among different prostheses design.

**Supplemental Figure 3.** **Bench top evaluation of differences in sizing and mitral bioprosthesis designs.** 25-mm, 27-mm, 29-mm, 31-mm and 33-mm manufacturer labeled mitral Epic, Mosaic, and Mitris bioprostheses are pictured. Row A in front shows the 25-mm bioprosthesis of the Epic, Mosaic and the Mitris with their respective larger devices sequentially lined up behind(33-mm devices are in the most distant field of view). Row B depicts the bench measurements taken of the distance between the struts anticipated to align to the anterolateral and anteroseptal trigones of the left ventricular outflow tract at the level of the sewing ring(white horizontal arrow), and again at the most ventricular portion of the 2 struts within the LVOT (orange horizontal arrow). Row C. Pictured is the variation in strut width design among the Epic, Mosaic and Mitral prostheses.

**Supplemental Video.** **Paravalvular leak in anterolateral trigone of Epic mitral bioprostheses.** Three of the four Epic mitral bioprostheses were noted to have a paravalvular leak at the anterolateral commissure of the mitral prosthesis sewing cuff post cardiopulmonary bypass epicardial echocardiographic interrogation. (See corresponding Figure 2.)
